# Long-range temporal organisation of limb movement kinematics in human neonates

**DOI:** 10.1016/j.cnp.2020.07.007

**Published:** 2020-08-14

**Authors:** Kimberley Whitehead, Judith Meek, Lorenzo Fabrizi, Beth A. Smith

**Affiliations:** a Department of Neuroscience, Physiology and Pharmacology, University College London, London WC1E 6BT, United Kingdom; b Elizabeth Garrett Anderson Wing, University College London Hospitals, London WC1E 6DB, United Kingdom; c Division of Biokinesiology and Physical Therapy and Department of Pediatrics, University of Southern California, Los Angeles, CA 90033, United States

**Keywords:** Motor, Somatosensory, Proprioception

## Abstract

**Objective:**

Movement provides crucial sensorimotor information to the developing brain, evoking somatotopic cortical EEG activity. Indeed, temporal-spatial organisation of these movements, including a diverse repertoire of accelerations and limb combinations (e.g. unilateral progressing to bilateral), predicts positive sensorimotor outcomes. However, in current clinical practice, movements in human neonates are qualitatively characterised only during brief periods (a few minutes) of wakefulness, meaning that the vast majority of sensorimotor experience remains unsampled. Here our objective was to quantitatively characterise the long-range temporal organisation of the full repertoire of newborn movements, over multi-hour recordings.

**Methods:**

We monitored motor activity across 2–4 h in 11 healthy newborn infants (median 1 day old), who wore limb sensors containing synchronised tri-axial accelerometers and gyroscopes. Movements were identified using acceleration and angular velocity, and their organisation across the recording was characterised using cluster analysis and spectral estimation.

**Results:**

Movement occurrence was periodic, with a 1-hour cycle. Peaks in movement occurrence were associated with higher acceleration, and a higher proportion of movements being bilateral.

**Conclusions:**

Neonatal movement occurrence is cyclical, with periods consistent with sleep-wake behavioural architecture. Movement kinematics are organised by these fluctuations in movement occurrence. Recordings that exceed 1-hour are necessary to capture the long-range temporal organisation of the full repertoire of newborn limb movements.

**Significance:**

Future work should investigate the prognostic value of combining these movement recordings with synchronised EEG, in at-risk infants.

## Highlights

• Movement occurrence is periodic in healthy newborns, with 1-hour cycle.
• Peaks in movement occurrence associated with higher acceleration, and higher proportion of movements being bilateral.
• Wearable sensors effectively characterise newborn movements, and have potential to be synchronised with EEG in future.

## 1 Introduction

In neonatal animals, motor activity and associated reafferent feedback is necessary for the development and refinement of sensorimotor thalamo-cortical circuits (Khazipov et al., 2004). In human neonates, self-generated and passive motor activity is also likely to be important for maturation of sensorimotor body maps, because movements evoke somatotopic cortical electroencephalography (EEG) and positive blood oxygen level–dependent responses in pre-term and full-term infants (Milh et al., 2007, Allievi et al., 2016, Losito et al., 2017, Whitehead et al., 2018, Whitehead et al., 2019a).

Human foetuses and pre-term and full-term infants exhibit two main patterns of motor activity: ‘writhing’ general movements in which the whole body participates, especially during wakefulness (Prechtl et al., 1997, Lüchinger et al., 2008), and isolated limb movements, especially during sleep (de Vries and Fong, 2006, Whitehead et al., 2018). Both movement types are thought to be generated by subcortical structures, that receive increasing descending modulation from first the subplate and then the cortical plate, potentially explaining their decline with development (Eyre et al., 2000, Rio-Bermudez et al., 2015, Inácio et al., 2016, Hadders-Algra, 2018).

In current clinical practice, neonatal motor activity is characterised by qualitatively examining the general movements occurring only during brief periods (a few minutes) of wakefulness (Thelen and Fisher, 1983, Gima et al., 2011, De Vries and Bos, 2011). Temporal-spatial organisation of these movements at the level of seconds, including short cycles of waxing and waning occurrence and acceleration, and diverse repertoire of limb combinations (e.g. unilateral progressing to bilateral), predicts positive sensorimotor outcomes (Prechtl et al., 1997, Einspieler et al., 2008). Recent work has demonstrated temporal-spatial organisation at the level of seconds for isolated limb movements also (Sokoloff et al., 2020). However, the *macro*-organisation of the *full* repertoire of neonatal motor activity, at the level of many minutes to hours, is unknown. This may be an important gap in knowledge, because long-range temporal organisation of the related neonatal parameter of EEG activity is the crucial anchor for all of its superimposed micro-organisation (Hartley et al., 2012). Here our objective was to quantitatively characterise the long-range temporal organisation of motor activity by monitoring kinematics over multi-hour recordings in a normative sample.

## 2 Methods

Eleven healthy full-term singleton newborns were recruited for this study from the postnatal (well baby) ward at the Elizabeth Garrett Anderson wing of University College London Hospitals (median 39 + 5 weeks + days corrected gestational age (range 38 + 5–41 + 2) and 1 day old (range 0.5–7); 5/11 female; median birth weight 3170 g). Ethical approval was obtained from the NHS Research Ethics Committee, and informed written parental consent was obtained prior to each study. The study complied with the 2013 update of the Declaration of Helsinki.

Limb movement was recorded using battery-powered sensors containing synchronised tri-axial accelerometers and gyroscopes (Opals v. 2, APDM), worn inside the pocket of custom-made ankle/wrist bands. All infants wore sensors on both legs, and 7/11 infants also wore sensors on both arms. Wearing the sensors did not change the quality of limb movements (video 1), in line with previous reports in infants (Jiang et al., 2018). Recordings lasted for a median of 3 h (range 2–4.5). This recording length was chosen because it typically includes a full sleep-wake behavioural cycle (Kleitman and Engelmann, 1953, Dittrichová, 1966, Stern et al., 1969, Stern et al., 1973, Anders and Roffwarg, 1973, Anders and Keener, 1985, Horne et al., 2001, Korotchikova et al., 2016). Each infant’s position and sleep-wake state were recorded by KW at the beginning and end of each recording. During the main part of the recording, the infants were left in private with their parents, in order to acquire naturalistic data non-intrusively.

**Supplementary figure 1:**
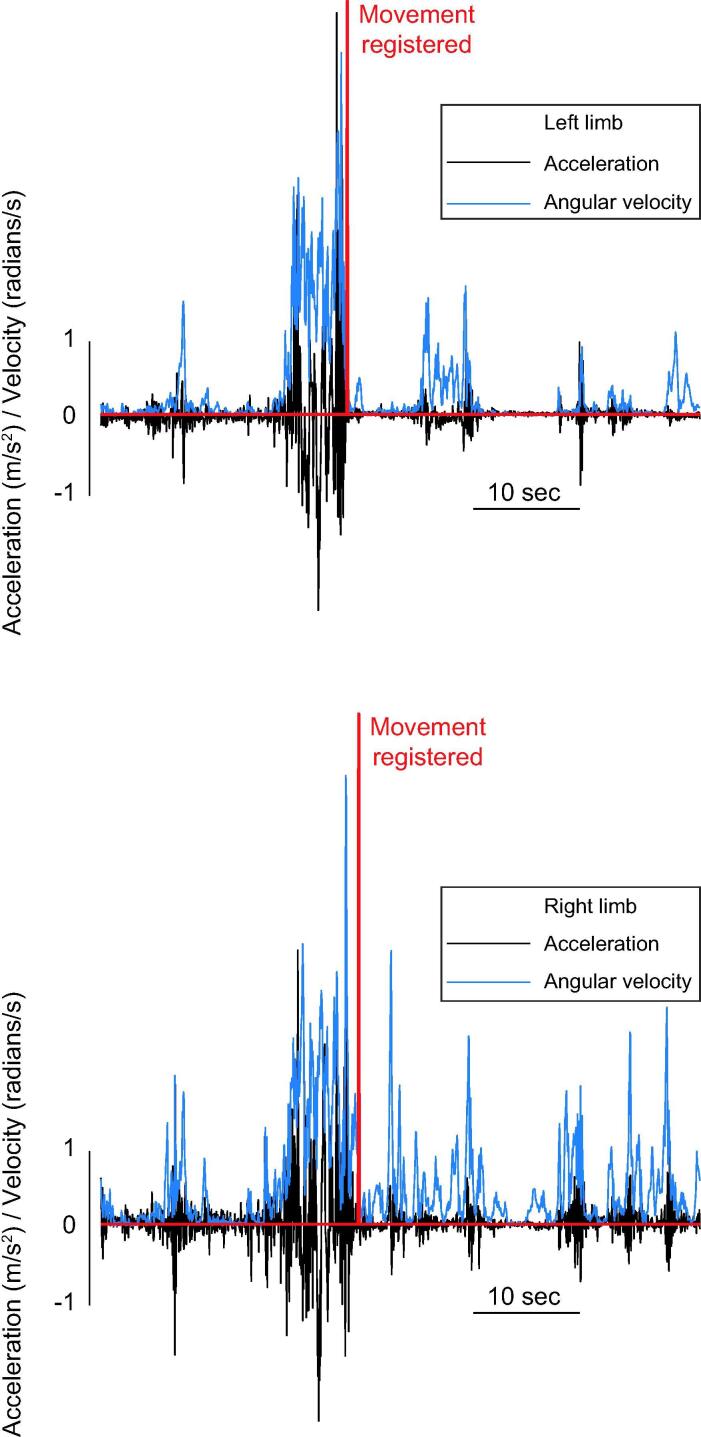
Identification of a bilateral limb movement. Example 1-minute long synchronised left and right limb acceleration and angular velocity traces from infant #3, within which a bilateral movement occurs between limbs.

Data were acquired at 128 Hz, and downsampled by a factor of 6 in order to use existing algorithms developed with 20 Hz data (Smith et al., 2015, Trujillo-Priego et al., 2017). Movements were identified when i) resultant acceleration (change in linear velocity per unit time) and ii) resultant angular velocity (change in limb orientation per unit time) passed a set threshold (Supplementary Fig. 1). Using resultant values considers overall magnitude, rather than direction, of acceleration/angular velocity. The advantage of analysing angular velocity in addition to acceleration is that this can highlight non-infant produced acceleration, e.g. a neonate being picked up, which should not be identified as a movement (Smith et al., 2015). Acceleration and angular velocity thresholds were identified separately for each individual recording, according to that recording’s variance. Movement identification was implemented using custom-written Matlab algorithms, previously validated in a separate sample of infants by video coding: for full details please see (Smith et al., 2015, Trujillo-Priego et al., 2017).

Leg vs. arm movement patterns have distinct qualities, e.g. the hip vs. shoulder joint has less range of motion, and therefore leg movements typically comprise discrete flexion–extension, while arm movements are more continuous. To take account of this, a separate leg movement was identified each time the leg paused or changed direction (Smith et al., 2015), while a separate arm movement was identified each time the arm paused (Trujillo-Priego et al., 2017). For this reason, the number of identified leg vs. arm movements cannot be directly compared: in infants who wore sensors on their legs and arms, there was a higher number of leg movements (median ratio 75:25), but these leg movements were shorter-lasting (p < .001 Mann-Whitney U Test) (Peirano et al., 1986, Cioni and Prechtl, 1990).

First, we examined how movements clustered in time across each recording. For this we used an algorithm which calculated the optimum number of clusters according to cohesion within-cluster (movements occurring in close temporal proximity), and separation between-clusters (cluster of movements occurs in isolation) (‘silhouette’ coefficient where 0.5–1 indicates good-quality clustering, maximum possible clusters set at 15, TwoStep algorithm, SPSS v. 26). Second, we assessed whether clustering of movement occurrence was periodic, by examining the spectral density of movement occurrence across each recording (movement counts per 5-minute bins).

Next, we investigated whether fluctuations in movement occurrence organised movement kinematics. Each movement was characterised by acceleration (mean acceleration across the sample points comprising the length of the movement), peak acceleration (highest acceleration value across the sample points comprising the length of the movement), and length (sample points comprising the movement). The leg movement algorithm also identified if a movement was bilateral, when movement of the homologous limb overlapped in time, even if not entirely synchronously (Supplementary Fig. 1). We tested for a relationship between movement count per 5-minute bin and i) median acceleration, peak acceleration, and length of movements within that bin (legs and arms pooled), and ii) proportion of movements which were bilateral within that bin, with Spearman’s correlations. Minimum 1 movement per 5-minute bin was necessary for inclusion in this analysis, in order to have a value for the kinematics indices. All statistical analysis was carried out using SPSS v. 26.

## 3 Results

29,165 movements were identified (23,679 leg; 5486 arm). The length of each movement ranged from 94 to 4836 msec (1st–99th centile).

**Fig. 1:**
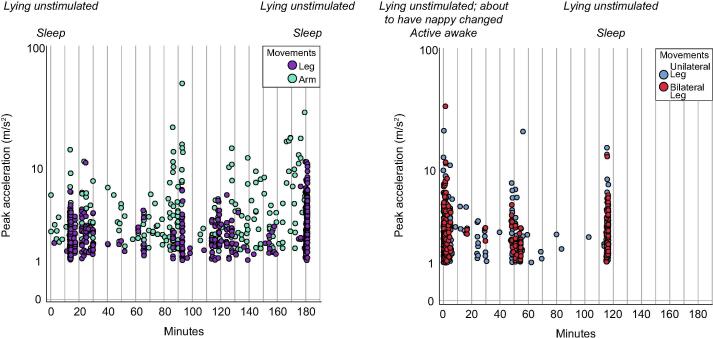
Clusters of movement occurrence. Left: Illustrative 3-hour time series of leg and arm movements from infant #5 (right + left limb pooled). For this subject, three clusters of leg movements were identified, and four clusters of arm movements were identified. Note that clusters of higher movement occurrence were associated with higher peak acceleration of movements. Right: Illustrative 2-hour time series of leg movements from infant #2 (right + left limb pooled) (plotted on a 3-hour x axis to facilitate comparison with the left panel). For this subject, three clusters of leg movements were identified. Note that clusters of higher movement occurrence were associated with a higher proportion of bilateral movements, and higher peak acceleration of movements, especially during a period of wakefulness at the beginning of the recording. The labels denote the infant’s position and sleep-wake state recorded by KW at the beginning and end of each recording.

**Fig. 2:**
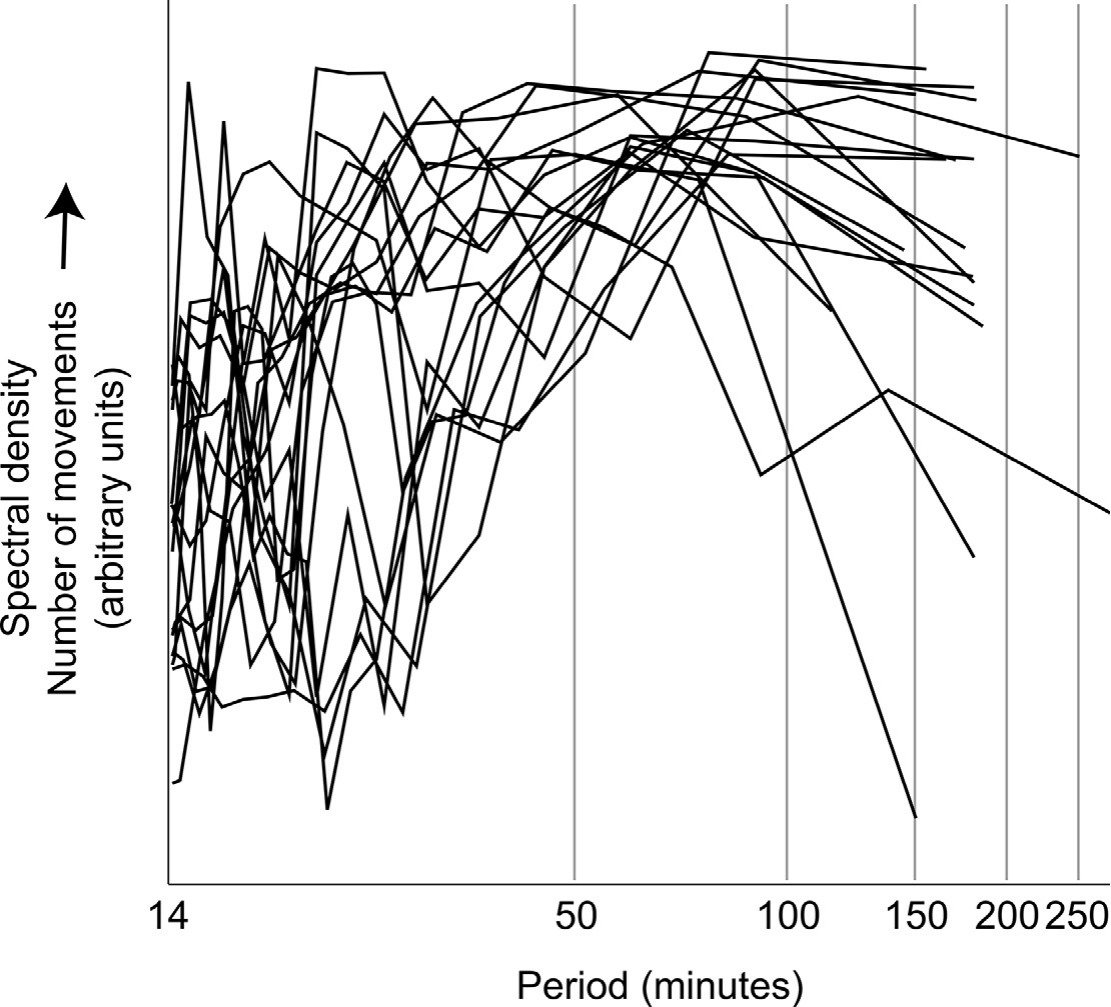
Periodicity of movement occurrence. Periodograms of number of movements for all 11 leg movement recordings and 7 arm movement recordings (right + left limb pooled) (smoothed using Tukey-Hamming window (span 15 min)). Traces differ in length because of the differing duration of each recording (range 2–4.5 h).

Clusters of movements were apparent in each infant’s time series, with associated fluctuations in acceleration (Fig. 1), and the proportion of movements which were bilateral (Fig. 1 right panel). A median of 3 clusters of movement occurrence were identified (min: 2, max: 5; all ‘silhouette’ coefficients >0.7). Spectral analysis indicated that these clusters of movements occurred periodically, with an approximately 60 min cycle observed consistently across recordings, and shorter cycles apparent in some instances (Fig. 2).

**Fig. 3:**
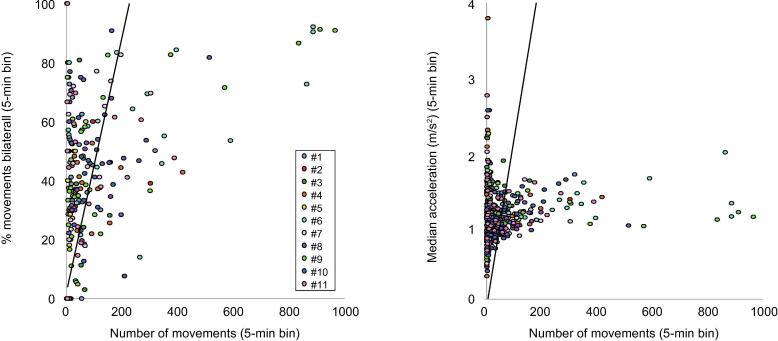
Movement kinematics were organised by fluctuations in movement occurrence. Higher movement occurrence was associated with a higher proportion of bilateral movements (left panel) and higher acceleration of movements (right panel).

Peaks in movement occurrence were associated with a higher proportion of movements being bilateral, and higher acceleration (proportion bilateral r. 548 p < .001, Fig. 3 left panel; acceleration: r .214 p < .001, peak acceleration: r .121 p = .005, Fig. 3 right panel). On the other hand, there was no relationship between movement occurrence and movement length (p = .439).

## 4 Discussion

Here we demonstrate for the first time that wearable sensors can be successfully used to quantitatively track movement kinematics over several hours in neonates who are just one day old. These unique multi-hour data allow to show that movement occurrence in neonates follows a 1-hour cycle, indicating that recordings must exceed one hour to capture the full repertoire of newborn movements. An additional novel result is that during peaks in movement occurrence, movements are more likely to be bilateral, and have higher acceleration.

**Supplementary figure 2:**
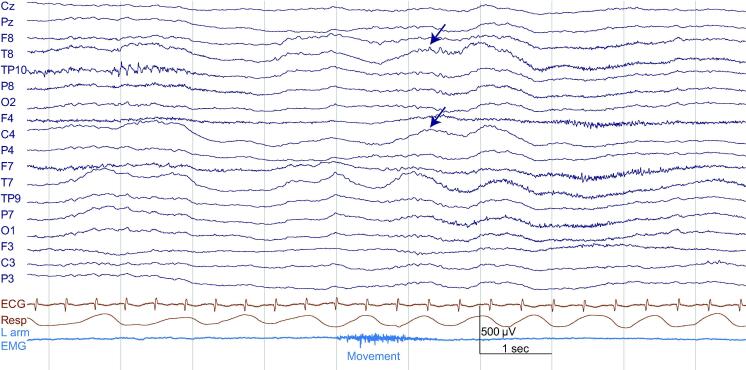
Illustrative example of contralateral brain oscillations following an isolated left arm movement during active sleep in a near-term infant. The arrows point to a localised increase in electroencephalography (EEG) oscillations post-movement overlying contralateral right sensorimotor (C4) and right mid-temporal (T8) regions. EEG filtered DC-70Hz; EMG filtered 0.5-800Hz (left flexor digitorum superficialis). ECG = electrocardiograph. Resp = respiratory movement. L = left. EMG = surface electromyograph.

At this maturational stage, isolated, unilateral limb movements are still evoking localised contralateral brain oscillations (Supplementary Fig. 2) (Whitehead et al., 2018), which refine thalamo-cortical circuits in neonatal animals (Khazipov et al., 2004). However, as infants’ unilateral cortical body maps near completion (Dall’Orso et al., 2018, Donadio et al., 2018), sensorimotor information associated with bilateral, high-density movements is also important, to strengthen inter- and intra-hemispheric connections (Whitehead et al., 2019b), ready for co-ordinated voluntary behaviours like reaching. Here we show for the first time that these distinct forms of sensorimotor information are cyclically organised at this critical juncture of development.

Higher movement acceleration has been associated with fine motor skill in older infants (Cahill-Rowley and Rose, 2018), but the higher the acceleration, the greater risk that targeted movements ‘overshoot’. Therefore, it may be advantageous for the developing brain to receive proprioceptive feedback associated with a wide repertoire of movement accelerations, as demonstrated here, to support calibration of emergent voluntary motor activity.

A 1-hour cycle of movement occurrence is consistent with sleep-wake behavioural architecture: an active-quiet sleep cycle lasts for 37–65 min at this age, from which wakefulness can arise (Kleitman and Engelmann, 1953, Dittrichová, 1966, Stern et al., 1969, Stern et al., 1973, Anders and Roffwarg, 1973, Anders and Keener, 1985, Curzi-Dascalova et al., 1988, Horne et al., 2001, Korotchikova et al., 2016). The shorter cycles apparent within this 1-hour cycle (Fig. 2) possibly indicate further heterogeneity *within* behavioural states (Peirano et al., 1986, Dvir et al., 2019, Whitehead et al., 2019c). While the same subcortical structures generate motor activity across sleep-wake states in neonatal animals, movement-initiating neural activity differs by state, potentially explaining the variance in motor phenotype putatively associated with state here (Rio-Bermudez et al., 2015, Inácio et al., 2016). Overall, our data imply a rich, highly variable repertoire of afferent input across a full behavioural cycle in human neonates. This may be one of the reasons that robust sleep-wake organisation is associated with healthy sensorimotor development (Osredkar et al., 2005, Shellhaas et al., 2017).

This study has some limitations. In particular, it was not possible to differentiate infant-generated movements from passive movements associated with caregiver interaction. However, infant-generated motor activity was robustly identified (many movements were identified during periods in which the infant was observed lying unstimulated, e.g. Fig. 1), and the length of identified movements is consistent with reports using other modalities to measure self-generated movement, such as electromyography (Supplementary Fig. 2) (Thelen and Fisher, 1983, Thelen, 1985, Hadders-Algra et al., 1993). Previous reports demonstrated that only around 15% of movements identified in infants using the present methodology are passive (Zhou et al., 2019). Inclusion of this small number of passive movements does not alter the significance of our findings, as both self-generated and passive motor activity play a role in sensorimotor development (Akhmetshina et al., 2016, Allievi et al., 2016), and the two will co-vary with behavioural state (e.g. infants typically wake to demand-feed, necessitating caregiver handling) (Georgoulas et al., 2020).

There are three other limitations of this study. Firstly, although the sample comprised a relatively homogenous sample of healthy newborn infants, the number of infants studied was small and future work should aim to replicate our findings in a larger cohort. Secondly, to definitively associate the periodicity of movement kinematics observed here with sleep-wake cycling will require future studies which include concurrent EEG and other polygraphic (e.g. respiratory) monitoring. Finally, while wearable sensors have the advantage of being non-intrusive, e.g. in comparison to electromyography which requires abrasive skin preparation, electromyography has its own advantages which include ability to differentiate individual muscle contributions.

In conclusion, here we demonstrate the rich diversity of the motor activity repertoire in healthy newborn infants, which fluctuates on an hourly cycle. Our quantitative methodology tracks movement with sufficient temporal resolution (sample points) that these kinematic time series could be convolved with synchronised EEG recordings in future studies. This would allow to investigate the value of combined multi-hour movement and cortical recordings for functional sensorimotor assessment of at-risk infants, such as those who sustained a brain injury (Whitehead et al., 2020).

## Appendix A Supplementary data

Supplementary video 1 Full-term infant aged 3 days old exhibiting general movements during active wakefulness. Note the comparability of limb movements between the first part of the video (not wearing sensors) and the second part of the video (wearing sensors on both legs and both arms inside ankle/wrist bands).
